# The initial effects of microclimate and invertebrate exclusion on multi-site variation in the mass loss of temperate pine and oak deadwoods

**DOI:** 10.1038/s41598-021-94424-w

**Published:** 2021-07-21

**Authors:** Seongjun Kim, Seung Hyun Han, Guanlin Li, Yujin Roh, Hyun-Jun Kim, Yowhan Son

**Affiliations:** 1 Center for Endangered Species, National Institute of Ecology, Yeongyang, Gyeongbuk Province 36531 Republic of Korea; 2 Institute of Life Science and Natural Resources, Korea University, Seoul, 02841 Republic of Korea; 3 Institute of Environment and Ecology, School of the Environment and Safety Engineering, Jiangsu University, Zhenjiang, 212013 China; 4 National Institute of Ecology, Seocheon, Chungnam Province 33657 Republic of Korea; 5 Department of Forest Resources, College of Agriculture and Life Science, Chonnam National University, Gwangju, 61186 Republic of Korea; 6 Department of Environmental Science and Ecological Engineering, Graduate School, Korea University, Seoul, 02841 Republic of Korea

**Keywords:** Ecology, Biogeochemistry

## Abstract

Quantifying deadwood decomposition is prioritized by forest ecologists; nonetheless, uncertainties remain for its regional variation. This study tracked variations in deadwood decomposition of Korean red pine and sawtooth oak in three environmentally different regions of the Republic of Korea, namely western, eastern, and southern regions. After 24 months, dead pine and oak woods lost 47.3 ± 2.8% and 23.5 ± 1.6% in the southern region, 13.3 ± 2.6% and 20.2 ± 2.8% in the western region, and 11.9 ± 7.9% and 13.9 ± 2.3% in the eastern region, respectively. The regional variation in the decomposition rate was significant only for dead pine woods (*P* < 0.05). Invertebrate exclusion treatment reduced the decomposition rate in all region, and had the greatest effect in the southern region where warmer climate and concentrated termite colonization occurred. The strongest influential factor for the decomposition of dead pine woods was invertebrate exclusion (path coefficient: 0.63). Contrastingly, the decomposition of dead oak woods was highly controlled by air temperature (path coefficient: 0.88), without significant effect of invertebrate exclusion. These findings reflect the divergence in regional variation of deadwood decomposition between pine and oak, which might result from the different sensitivity to microclimate and decomposer invertebrates.

## Introduction

Deadwoods are significant in forest ecology, given their crucial roles in forest ecosystem functions^1^. They participate in terrestrial carbon budgets to store dead organic carbon and serve as a carbon dioxide efflux source^2–4^. The presence and decomposition of deadwoods also stimulate pedogenic processes by differentiating soil humidity from the surrounding areas and incorporating additional organic substrates into the soil^5,6^. Deadwoods provide feeding sources and microhabitats for soil fauna and flora, and thus, contribute to biodiversity conservation^7^.

Tracking the controlling factors of decomposition rate is a prioritized topic in deadwood studies. From this traditional perspective, microclimatic conditions dominate deadwood decomposition^1^. Temperature controls the decomposer communities in deadwoods and the surrounding environments, and strongly mediates the microbial degradation of organic carbon sources into carbon dioxide^8,9^. Alternatively, moisture is considered a counteracting factor against the stimulating effect of temperature because the deadwood decomposition can be hindered under excessive humidity and drought^10,11^. Such microclimatic effects can vary with woods' physical and chemical composition, the primary trait for each tree species^12^. These aspects have enabled forest ecologists to simulate the carbon dynamics using the species-specific average decay constants, modified by the temperature-dependent gradients (e.g., Q_10_ values) and the moisture functions^13,14^.

Several studies have reported the importance of uncertainties left behind spatial variations in the deadwood decomposition rate. In particular, Bradford et al*.*^14^ suggested that simple modification using microclimate possibly fails to represent local variation in deadwood decomposition due to spatial inconsistency in decomposer abundance, although it can predict the global patterns effectively. Shorohova and Kapitsa^15^ also reported that the deadwood decomposition could differ throughout European boreal forests because of regional inconsistency in site fertility and tree species as well as microclimatic conditions. Similar spatial variabilities have been detected in microsite levels from landscape-scale studies^16–18^. The simple aggregation of such variations into an average value may reduce overall accuracy of estimating deadwood dynamics and associated ecosystem processes^14^. Considering the limited knowledge about deadwood ecology, elucidating the decomposition variability of deadwoods can aid to understanding of forest sciences and efforts to predict forest carbon and nutrient budgets^19^.

Decomposer invertebrates like termites and beetles may affect the variation in deadwood decomposition^20^. The colonization of wood decomposing invertebrates potentially creates hot-spots showing a faster decomposition rate relative to the area without such species^20,21^. The invertebrates sometimes distort the climatic gradients in deadwood decomposition rate owing to their tolerance to the excessively harsh conditions for microbial decomposers^22^. Such effects of invertebrates may interact with the influences of wood species traits and abiotic environments, given the diversity in their feeding and habitat preferences^12,23^. However, most of these findings have focused chiefly on tropical and subtropical zones^24^, while the tendencies underlying the other climatic areas were often underrated despite some empirical evidence^21,25,26^.

**Figure 1:**
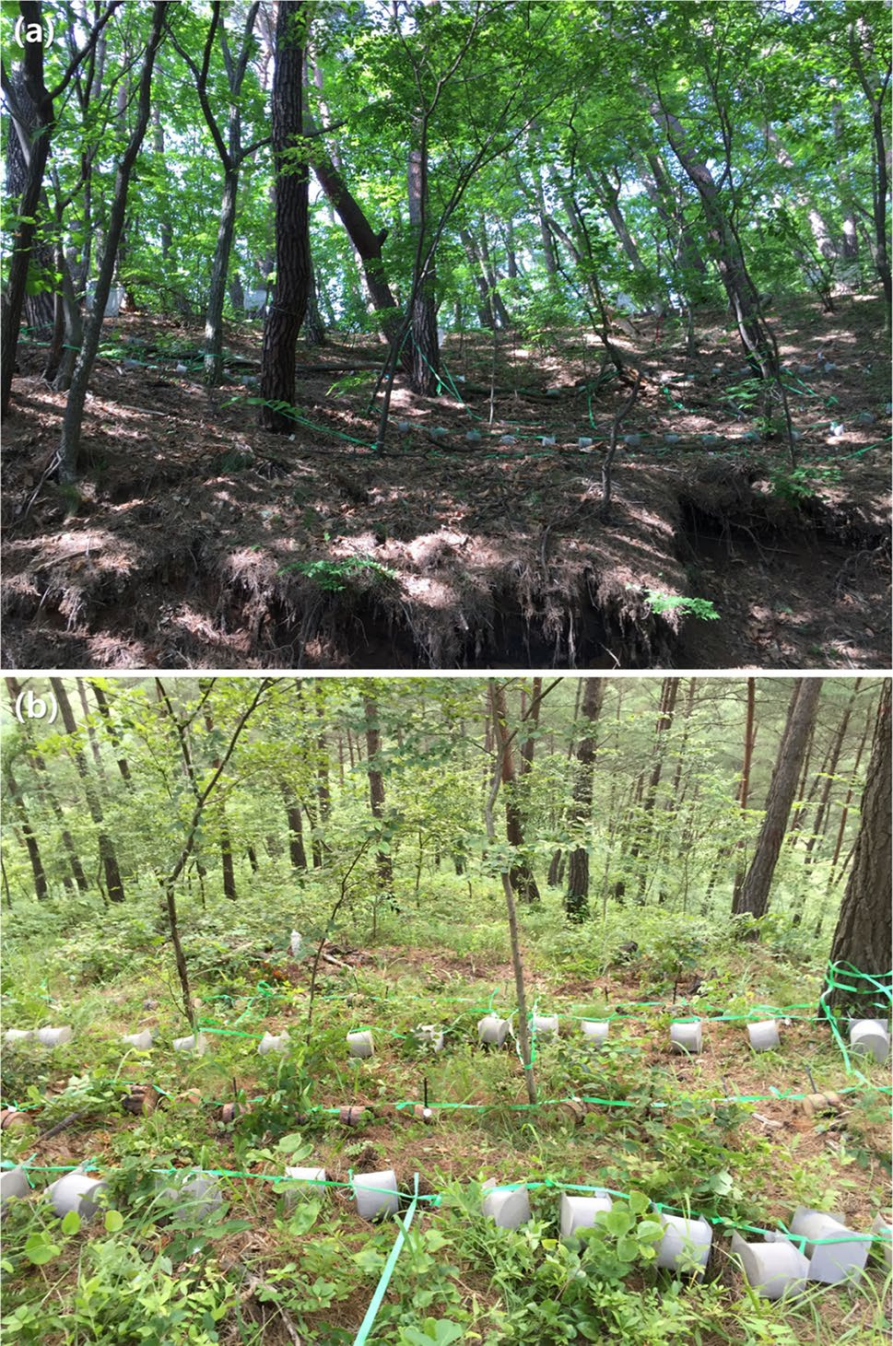
Photographs of the initial deadwood samples in site 1 (**a**) and site 8 (**b**) (photograph by Seongjun Kim).

The present study aimed at the variation in the deadwood decomposition of Korean red pine (*Pinus densiflora* Sieb. et Zucc.) and sawtooth oak (*Quercus acutissima* Carruth). These tree species are common in both plantations and naturally regenerated forests of the cool and warm temperate climatic zones in Northeast Asia^27^. The primary hypothesis is that the variation in deadwood decomposition rate would occur across sites depending on the differences in environmental conditions (temperature, humidity, and soils) and decomposer invertebrate activities. This hypothesis was based on the facts that environmental conditions can control the decomposers’ physical (fragmentation) and chemical (respiration) decay processes^11,28^ and the areas with a high incidence of decomposer invertebrate can feature a relatively fast wood decomposition rate than the other areas^20,21^. To prove this hypothesis, pine and oak deadwoods were experimentally set to the nine study sites at the three environmentally distinct regions (western, eastern, and southern) in the Republic of Korea (Fig. 1). The mass loss rate of deadwoods was monitored at each study site for 24 months. Moreover, the mesh enclosing treatment was implemented in half of the deadwood samples, which has been broadly used to examine soil invertebrates' contributions to wood decomposition processes^8,29–31^.

## Results

### Deadwood decomposition patterns

**Figure 2:**
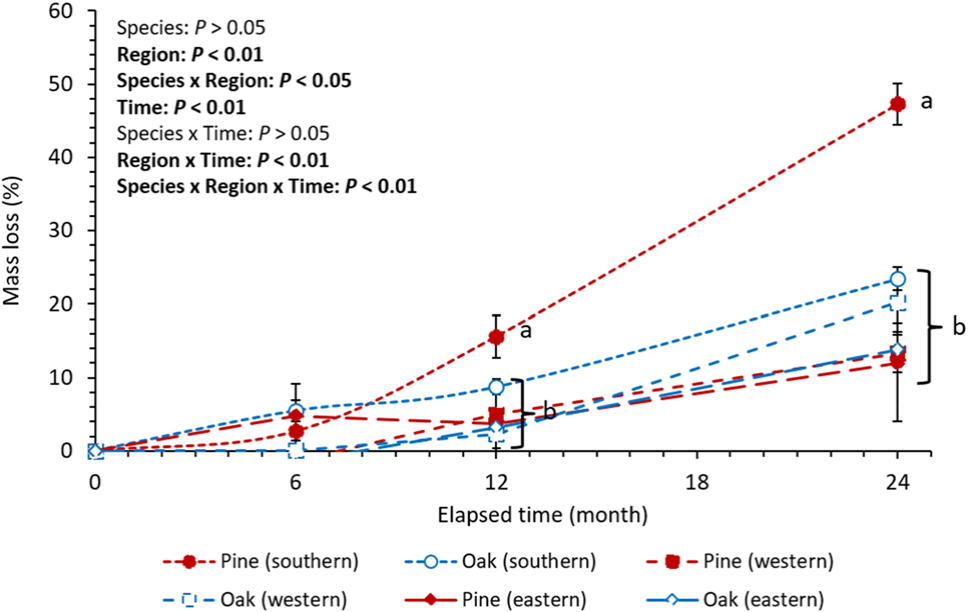
Mass loss of deadwood samples within the study period (24 months). Letters next to points show statistical differences between the mass losses within a given time (n = 9 for each species within a given region and time, *P* < 0.05). Vertical bars indicate standard errors.

Pine and oak deadwood samples lost, on average, 24.2 ± 4.3% (k = 0.138 ± 0.017 year^−1^) and 19.1 ± 1.5% (k = 0.106 ± 0.005 year^−1^) of the initial mass throughout all regions after 24 months. Nevertheless, the mass loss rate was highly variable among regions. Mass loss after 24 months was 47.3 ± 2.8% (k = 0.320 ± 0.027 year^−1^), 13.3 ± 2.6% (k = 0.071 ± 0.015 year^−1^), and 11.9 ± 7.9% (k = 0.064 ± 0.045 year^−1^) for pine, and 23.% ± 1.6% (k = 0.134 ± 0.011 year^−1^), 20.2 ± 2.8% (k = 0.113 ± 0.018 year^−1^), and 13.9 ± 2.3% (k = 0.075 ± 0.013 year^−1^) for oak in the southern, western, and eastern regions, respectively (Fig. 2). The general linear mixed model exhibited that the effects of region and time were significant (*P* < 0.01), whereas tree species alone had no significant effect on deadwood mass loss (Fig. 2). Significant interaction effects were detected from the combinations of species and region (*P* < 0.05), region and time (*P* < 0.01), and species, region, and time (*P* < 0.01) (Fig. 2). The post-hoc test reported that the mass loss of pine deadwoods was significantly higher in the southern region than in the western and eastern regions after 12 and 24 months (*P* < 0.05) (Fig. 2). Mass loss of oak deadwoods, however, did not significantly differ among the three regions, even though the average values presented a similar tendency with pine deadwoods (Fig. 2). The difference in mass loss between pine and oak was significant only in the southern region after 12 and 24 months (*P* < 0.05) (Fig. 2).

**Figure 3:**
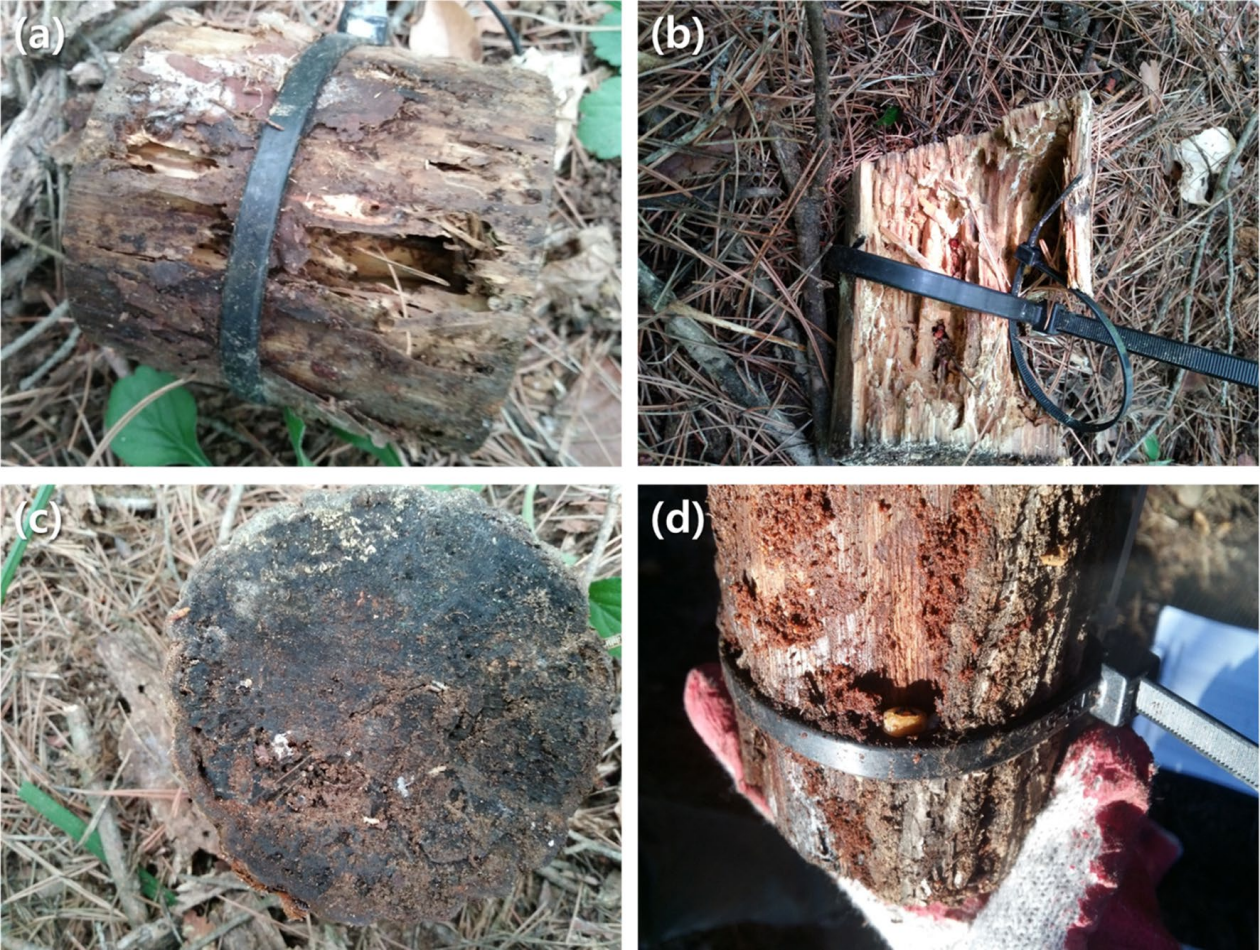
Photographs of decomposer invertebrates on deadwood samples. Feeding damages by subterranean termites on pine (**a**, **b**) and oak (**c**) samples, and incidence of beetle larvae (**d**) after 24 months (photograph by Seongjun Kim).

Deadwood samples exhibited visible damages from invertebrates, including subterranean termites (Fig. 3a–c) and beetles larvae (Fig. 3d). The damage from subterranean termites (*Reticulitermes speratus kyushuensis* Morimoto) was common and highly intensive in the southern region (Fig. 3a–c), but such pattern did not occur in the western and eastern regions. Though the subterranean termites attacked both tree species in the southern region, pine deadwoods (Fig. 3a, b) received more severe visible damage than oak deadwoods (Fig. 3c). Conversely, the damages from the other decomposer invertebrates (e.g., *Neocerambyx raddei* Blessig, *Tomicus piniperda* Linnaeus, *Platypus koryoensis* Murayama, *Scolytoplatypus tycoon* Blandford, *Dorcus titanus castanicolor* Motschulsky, and *Dorcus rectus rectus* Motschulsky) were not as intensive as those from termites but widespread across tree species and regions (Fig. 3d). As intended, the invertebrate exclusion treatment prevented the access of invertebrates, and no visible invertebrate damage was found on the samples enclosed with mesh during the study period. The invertebrate exclusion effect was negative for all species and study sites, indicating that the exclusion treatment reduced deadwood mass loss in all regions (Table 1). Invertebrate exclusion generally had a greater effect size in the southern region than in the western and eastern regions (Table 1), which was in agreement with the visible damages from subterranean termites.

**Table 1:** The invertebrate exclusion effect and invertebrate damage on deadwood samples in each study site.

Region	Study site	Invertebrate exclusion effect^a^	
		Pine	Oak
Western	Site 1	0.42 ± 1.63	0.68 ± 1.67
	Site 2	0.71 ± 1.67	1.08 ± 1.77
	Site 3	2.23 ± 2.22*	0.85 ± 1.71
Eastern	Site 4	0.27 ± 1.61	4.42 ± 3.46*
	Site 5	0.60 ± 1.65	1.34 ± 1.85
	Site 6	0.29 ± 1.61	0.44 ± 1.63
Southern	Site 7	3.79 ± 3.07*	1.30 ± 1.84
	Site 8	4.94 ± 3.78*	4.74 ± 3.65*
	Site 9	2.77 ± 2.50*	2.84 ± 2.54*

^a^Effect size based on Hedges’ *d* ± 95% confidence interval. Higher value indicates the larger decrease in mass loss of deadwood samples due to the invertebrate exclusion. Values with asterisk are statistically significant at *P* < 0.05 (the 95% confidence interval did not overlap with zero).

### Influential factors for the decomposition rate

**Figure 4:**
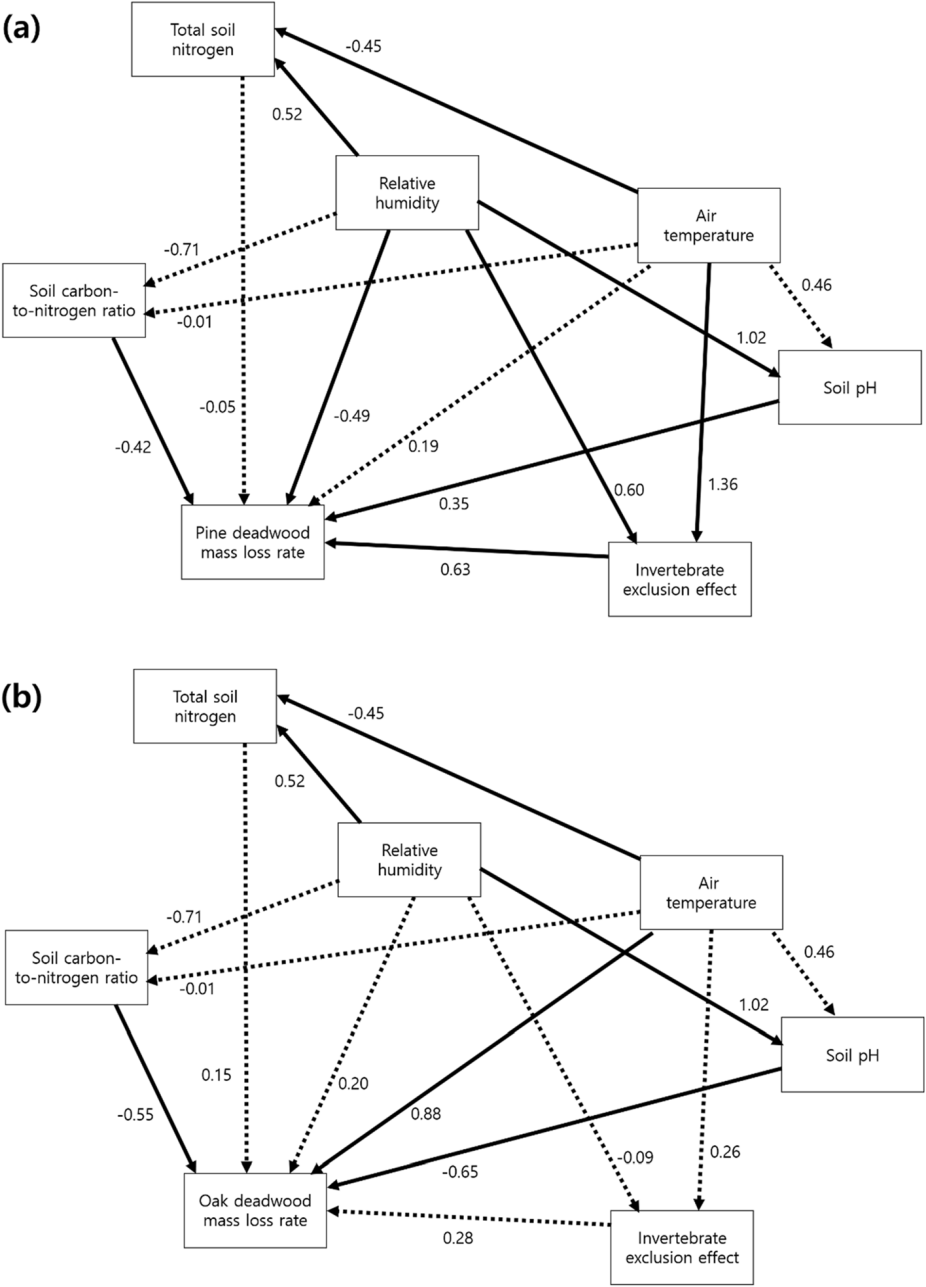
Results of structural equation models for the mass loss of pine (**a**) and oak (**b**) deadwoods. Values next to each arrow show path coefficients. Straight and dashed arrows indicate significant and non-significant effects at *P* < 0.05.

The structural equation model for pine showed that invertebrate exclusion effect was the strongest factor directly affecting the mass loss of pine deadwoods, with a path coefficient of 0.63 (Fig. 4a). Although air temperature had no significant effect on the mass loss of pine deadwoods, it indirectly contributed to the mass loss rate as the strongest influential factor for invertebrate exclusion effect (Fig. 4a). Conversely, the structural equation model for oak demonstrated that air temperature was the strongest factor affecting the mass loss of oak deadwoods, with a path coefficient of 0.88 (Fig. 4b). Invertebrate exclusion effect was not a significant influential factor of the mass loss of oak deadwoods (Fig. 4b), which was on the contrary to the pattern in pine deadwoods.

**Figure 5:**
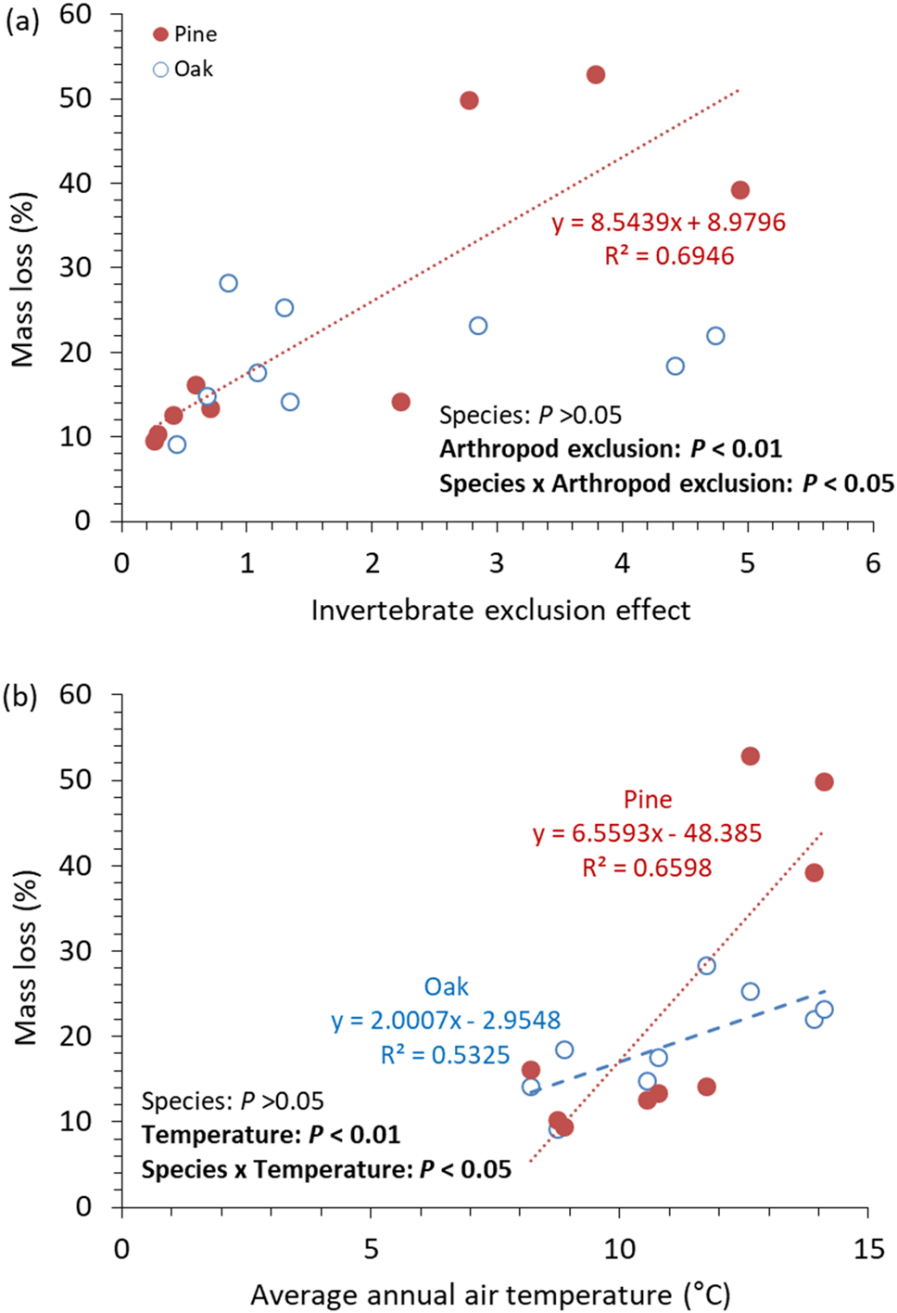
Relationships of mass loss of deadwood samples after 24 months to invertebrate exclusion effect (**a**) and average annual air temperature (**b**). Only significant regression lines are presented on each panel (n = 9, *P* < 0.05). Dots in each panel represent the average value from three plots within each study site. The higher invertebrate exclusion effect indicates the larger decrease in the mass loss rate of deadwood samples by invertebrate exclusion.

The univariate tests using the strongest influential factors for pine (invertebrate exclusion effect) and oak (air temperature) deadwoods reported that deadwood samples differed in sensitivity to air temperature and invertebrate exclusion (Fig. 5). The analysis of covariance demonstrated significant interaction effects between species and either invertebrate exclusion or air temperature (*P* < 0.05), and the slopes of the linear regression lines were steeper for pine deadwoods than for oak deadwoods (Fig. 5). These results suggest that oak deadwood decomposition was more tolerant to the gradient of air temperature and decomposer invertebrate activity than pine deadwood decomposition, which possibly led to the relatively low regional variation in the mass loss of oak deadwoods (Fig. 2).

## Discussion

The mass loss rate of pine deadwoods was significantly higher in the southern region than in the other regions (*P* < 0.05, Fig. 2). Results also demonstrate that invertebrate exclusion effect was the most influential factor for the variation in the mass loss of pine deadwoods (Fig. 4a). This pattern agrees with case studies that have reflected the regional difference in pine deadwood decomposition between central (k = 0.086–0.097 year^−1^)^32,33^ and southern regions (k = 0.267–0.506 year^−1^)^34^. Our results fit into previous studies, emphasizing wood decomposition mechanisms by decomposer invertebrates, including direct feeding and enzymatic digestion, wood structural change owing to tunneling and fragmentation, and biotic interaction with microbial community composition and activity^35^. Though microbial data were not available in the present study, tunnels and fragmentation by termites prevailed in the southern region only (Fig. 3a–c) as displayed in the invertebrate exclusion experiment by Ulyshen et al.^26^ for *Pinus taeda* L. logs. Such concentrated physical damage by decomposer invertebrates in the southern region might contribute to the regional difference in invertebrate exclusion effect (Table 1), and consequently intensify the variation in the mass loss of pine deadwoods as the strongest influential factor (Fig. 4a). This result is similar to Warren and Bradford^20^, who reported a significant increase in the mass loss of artificial wooden nests at the temperate mixed hardwood forests with termite colonization. Therefore, the results of the present study support the potential role of decomposer invertebrate activity in the spatial variation in temperate pine deadwood decomposition, as indicated in the tropical wet and dry environments^17,21,22,36,37^.

The mass loss of oak deadwoods, in contrast to pine deadwoods, exhibited only marginal regional variation (Fig. 2) and were unrelated to invertebrate exclusion effect (Figs. 4b, 5a). It was also found that air temperature was the strongest factor directly influencing the mass loss of oak deadwoods, but it was not related to invertebrate exclusion effect for oak (Fig. 4b). These results imply that oak deadwood decomposition might respond to the temperature-related decay mechanism except for an indirect process through decomposer invertebrate activity^8^. In the present study, oak samples were higher both in initial wood nitrogen concentration (pine: 0.05 ± 0.01%, oak: 0.11 ± 0.00%) and density (pine: 0.4 ± 0.0 g cm^−3^, oak: 0.6 ± 0.0 g cm^−3^) than pine samples. Such initial condition of oak deadwoods could be favorable to chemical decomposition by microbes to acquire nitrogen^38^, rather than invertebrate activity because of dense microfibril arrangement^23^. This is consistent with an indoor experiment by Yoon et al.^39^, who reported that microbial respiration from oak deadwoods was higher than that from pine deadwoods, and more sensitive to the surrounding temperature condition. Accordingly, our results indicate that the mass loss of oak deadwoods might correspond to the microbial decomposition level along the air temperature gradient, and be less sensitive to invertebrate activity than pine deadwood decomposition.

It is important to note that air temperature strongly affected invertebrate exclusion effect on pine deadwoods, the strongest influential factor for pine deadwood decomposition (Fig. 4a). Also, air temperature was positively related to the mass loss of pine deadwoods (Fig. 5b), despite no significance in the direct air temperature effect on pine deadwood decomposition (Fig. 4a). Although several studies suggested that the activity of decomposer invertebrate could confound the patterns in deadwood decomposition along a climatic gradient^37^, such tendency was not detected in the present study. These findings enable us to expect that air temperature might be important for pine deadwood decomposition as an indirect factor controlling decomposer invertebrate activity.

The general linear model revealed that there was a significant difference in regional variation in the mass loss rate between pine and oak deadwoods (interaction effect of region and species, Fig. 2). Mechanisms for such relationship between regional variability and tree species remain unclear due to the limited number of relevant literatures. However, our results show that only the mass loss of pine deadwoods was significantly correlated to the invertebrate exclusion effect (Fig. 5a), and both concentrated termite damage and atypically high mass loss of pine deadwoods occurred in the southern region rather than the western and eastern regions (Fig. 2). This pattern implies that inconsistent sensitivity to decomposer invertebrates possibly led to the different regional variations between pine and oak deadwoods. In fact, the differences in feeding preference are not uncommon in other termite studies^12,40,41^, and termites’ feeding preferences could intensify the effect of tissue density and recalcitrant compound content on wood decomposition rate^23^. Furthermore, the detected subterranean termite species, *R. speratus kyushuensis* is known to prefer *P. densiflora* than broadleaf tree species as a feeding source because of the difference in wood density and recalcitrant chemical content^28^. Oak deadwoods used in the present study actually had a higher wood density than pine deadwoods, and thus, could be more tolerant to physical damage by invertebrate decomposers^19^. Such preferences for pine deadwoods might create a significant difference between the mass losses of pine and oak deadwoods in the southern region accordingly. Inversely, the differential effects on tree species were not notable in the western and eastern regions because of the absence of termite feeding damages and the weaker invertebrate exclusion effect (Table 1), which might cause more distinct regional variation in deadwood decomposition for pine relative to oak. This explanation agrees with the binomial elevation of wood decomposition rate depending on whether *Reticulitermes* termite colonization exists or not in Warren and Bradford^21^.

This study’s findings should be carefully interpreted as our study period was limited only for 24 months. Deadwoods are traditionally considered to require an extended period of the decomposition process^1^. Previous studies have also reported either an increase^26^ or a decrease^40^ in the contribution of invertebrates to deadwood decomposition with the time frame. Hence, further monitoring should be followed to ensure if the divergence in the regional variation between pine and oak deadwood decomposition persists in the long term.

Overall results support our primary hypothesis on the regional variation in deadwood decomposition, which was mainly related to invertebrate activity (pine) and air temperature gradient (oak). However, pine deadwoods had a larger regional variation in the mass loss rate than oak deadwoods owing to the concentrated of decomposer invertebrates in the southern region and the higher sensitivity to such decomposer activity. This finding suggests that not only the temperature gradient, but distribution and activity of decomposer invertebrates might reshape the regional variation in deadwood decomposition among multiple temperate forest ecosystems. In terms of forest ecology, decomposing deadwoods are important sources of organic carbon and nutrients, which could result in spatial divergence in quality and fertility of the surrounding soil environments^2,5^. Therefore, the detected variation among regions and the two species might reflect the associated regional variability of the soil environment as well as tree growth, forest productivity, and carbon dynamics^42^. The subsequent long term monitoring should be followed to explore such aspect, considering the slow progression of deadwood decomposition.

## Methods

### Study sites

The present study targeted three environmentally different regions in the Republic of Korea, namely western, eastern, and southern regions (Table 2). The regions had under a temperate climate with a concentrated rainfall during summer and cold, dry winter. However, the southern region featured a warmer microclimate and lower altitude than the other regions, while the eastern region had a colder, moister microclimate and nitrogen rich soils (Table 2). The western region is characterized by an intermediate environment between the southern and eastern regions, although it contained the most acidic soils (Table 2). Such differences in microclimate and soils are expected to differentiate decomposer activity and deadwood mass dynamics across the regions^42,43^.

**Table 2:** Environmental conditions in the study sites in the western, eastern, and southern regions.

Region	Site	Location	Stand density (stem ha^−1^)	Altitude (m)	Air temperature^a^ (°C)	Relative humidity^a^ (%)	Soil pH	Total soil N^b^ (g kg^−1^)	Soil C/N ratio^b^	Major tree species^c^
Western	Site 1	37°47′N, 127°10′E	650	420	10.56	73.56	4.64	1.20	28.58	*Pinus densiflora*, *Quercus mongolica*, *Quercus serrata*, *Acer pseudosieboldianum*
	Site 2	37°46′N, 127°10′E	1140	470	10.77	73.13	4.43	1.11	30.53	*P. densiflora*, *Q. mongolica*, *Q. serrata*, *A. pseudosieboldianum*
	Site 3	37°44′N, 127°10′E	2500	500	11.73	75.54	4.42	1.19	26.77	*P. densiflora*, *Q. serrata*, *A. pseudosieboldianum*
Eastern	Site 4	37°30′N, 128°56′E	610	690	8.88	77.10	5.10	1.78	22.71	*P. densiflora*, *Quercus aliena*, *Quercus variabilis*, *Acer pictum* subsp. *mono*
	Site 5	38°02′N, 128°22′E	1050	610	8.21	81.73	5.17	2.49	18.93	*P. densiflora*, *Q. mongolica. Carpinus cordata*
	Site 6	38°03′N, 128°22′E	900	590	8.74	81.60	5.21	1.69	21.23	*P. densiflora*, *Q. mongolica. C. cordata*
Southern	Site 7	35°21′N, 128°10′E	1200	430	12.61	74.56	5.07	0.97	24.56	*P. densiflora*, *Q. variabilis*, *A. pictum* subsp. *mono*, *Toxicodendron vernicifluum*
	Site 8	35°12′N, 128°10′E	600	170	13.90	72.83	4.77	1.02	35.42	*P. densiflora*, *Quercus dentata*, *Quercus acutissima*
	Site 9	35°12′N, 128°10′E	1560	180	14.11	71.64	4.89	0.64	23.44	*P. densiflora*, *Q. dentata*, *Q. acutissima*, *T. vernicifluum*

^a^The average annual values during the study period (24 months).

^b^Total soil N: total soil nitrogen; soil C/N ratio: soil carbon-to-nitrogen ratio.

^c^Tree species dominating canopy and sub-canopy layers.

Nine study sites were selected for the deadwood decomposition experiment. Three study sites were included for each region (western region: sites 1–3; eastern region: sites 4–6; southern region: sites 7–9) (Table 2). All study sites were located in the stands with the Korean red pine dominating canopy and broadleaf species (e.g., *Quercus* spp.) dominating mid-to-understory layers, which is the most common forest type in the country^44^. Study sites in the same region shared similar average annual air temperature, relative humidity, and soil properties, but differed in stand density (Table 2).

### Deadwood decomposition experiments

Deadwoods for the decomposition experiment were collected from a mixed Korean red pine and sawtooth oak stand in Bonghwa, Republic of Korea. All the deadwood samples were thinned and harvested at a single site in March 2016 (2 months before the decomposition experiment) to focus on the regional variation in decomposition rate without heterogeneity in the initial deadwood conditions, such as decay class and soil nutrient, microclimate, and tree composition of the sampling site. The sample trees were also similar in age (20-year-old) and processed into the consistent cylindrical shape to reduce unexpected confounding influences (Fig. 1). Initial dry mass, diameter, length, wood density, carbon concentration, and nitrogen concentration were 361.4 ± 3.5 g, 11.1 ± 0.1 cm, 10.2 ± 0.0 cm, 0.4 ± 0.0 g cm^−3^, 48.9 ± 0.3%, and 0.05 ± 0.01% for pine deadwood samples and 427.7 ± 5.0 g, 9.4 ± 0.1 cm, 9.9 ± 0.0 cm, 0.6 ± 0.0 g cm^−3^, 48.4 ± 0.2%, and 0.11 ± 0.00% for oak deadwood samples, respectively. In total, 972 deadwood samples were prepared for each tree species.

Three plots (4 m × 10 m) were established in each study site to clarify the decomposition experiment area (Fig. 1, 27 plots in total). The plots within the same study site had a similar environment (e.g., topography and tree species composition) and were buffered by a 5–10 m wide strip area to avoid uncertainties by spatial distribution. In May 2016, 72 deadwood samples were allocated for each plot (36 samples × 2 tree species), and were fastened horizontally on a slope using strings and cable ties after removing the top litter and humus layer. Half of the deadwood samples were enclosed by a 0.26 mm stainless mesh screen to exclude the access of decomposer invertebrates, such as termites and beetles (Fig. 1). Similar mesh pore sizes have been used by the wood decomposition experiments aiming to eliminate the damage by soil micro- and meso-invertebrates^8,18,29–31^.

Fresh mass of all deadwood samples without invertebrate exclusion was quantified 6, 12, and 24 months after the plot establishment on each site. One pine and oak deadwood samples were collected from each plot and dried at 85 °C to determine the fresh-to-dry mass ratio. The dry mass of deadwood samples was estimated using fresh mass and fresh-to-dry mass ratio. Mass loss was calculated based on the percentage of decrease in the estimated dry mass relative to the initial dry mass. The decay constant (year^−1^) during the entire study period was also calculated to ease comparisons with the previous deadwood studies as follows;

$$ {\text{Decay}}\,{\text{ constant}}\, \left( {\text{k}} \right) = \ln \left( {\frac{mass\, remaining\, after\, 24\, months}{{initial \,mass}}} \right) \div 2 \,years $$

Meanwhile, the final dry mass of deadwood samples with invertebrate exclusion was measured after 24 months to analyze the invertebrate exclusion effect (see statistical analyses subsection). Visible damage by invertebrate feeding on the deadwoods was recorded simultaneously.

### Environmental data collection

Microclimate data (i.e., air temperature and relative humidity) were monitored every 1 h with an air temperature and humidity sensor (U23-001, Onset, USA) until 24 months after the experiment initiation. A temperature and humidity sensor was installed 30 cm above the soil surface in each study site. Between May and June 2017, mineral soil at 0–10 cm depth was sampled from 15 random locations within each study site, and was incorporated into one composite sample per study site. The soil samples were air-dried and sieved with a 2-mm mesh screen before use for soil analyses. Soil pH was measured under a 1:5 soil-to-water ratio using a refillable electrode (ROSS Ultra pH/ATC Triode, Thermo Scientific Orion, USA). Total soil carbon and nitrogen concentrations were determined using an elemental analyzer to calculate soil carbon-to-nitrogen ratio (vario Macro, Elementar Analysensysteme GmbH, Germany).

### Statistical analyses

The invertebrate exclusion effect on deadwood decomposition was quantified and standardized according to Hedges’ *d* and a 95% confidence interval^45,46^, as applied by the wood decomposition experiment of Ulyshen^31^;

$$ {\text{Invertebrate }}\,{\text{exclusion}}\,{\text{ effect}} = { }\frac{{ML_{unexcluded} - ML_{excluded} }}{{S_{pooled} }} \times \left[ {1 - \frac{3}{{4\left( {n_{unexcluded} + n_{excluded} - 2} \right) - 1}}} \right] $$

$$ S_{pooled} = { }\sqrt {\frac{{\left( {n_{unexcluded} - 1} \right)S_{unexcluded}^{2} + \left( {n_{excluded} - 1} \right)S_{excluded}^{2} }}{{n_{unexcluded} + n_{excluded} - 2}}} $$

where ML_unexcluded_ and ML_excluded_ represent the average mass loss of deadwoods without and with invertebrate exclusion after 24 months, and n_unexcluded_ and n_excluded_ denote the number of plots. S_unexcluded_ and S_excluded_ are standard deviations for the mass loss of deadwoods without and with invertebrate exclusion. The positive invertebrate exclusion effect means the deceleration of the mass loss rate of deadwoods by the invertebrate exclusion. The 95% confidence interval for the invertebrate exclusion effect was estimated as follows^45,46^;

$$ 95{\text{\% confidence }}\,{\text{interval}} = { } \pm { }1.96\sqrt {\frac{{n_{unexcluded} + n_{excluded} }}{{n_{unexcluded} \times n_{excluded} }} + \frac{{invertebrate\, exclusion\, effect^{2} }}{{2\left( {n_{unexcluded} + n_{excluded} - 2} \right)}}} $$

The general linear mixed model was conducted to compare deadwood mass loss among the tree species and regions along the time frame. This model comprised the fixed effects of tree species and region and the random effects of elapsed time and the study site within a region (block) on the mass loss of deadwood samples. A post-hoc Tukey’s HSD test followed this general linear mixed model, and a plot within each study site was used as the unit of replication (n = 9 for each tree species in each region at a given time frame; *P* < 0.05). The values were log-transformed for this analysis to achieve the normality criteria. The general linear mixed model was conducted with the proc glimmix procedure in SAS v.9.4 software (SAS Institute Inc., USA).

Structural equation model was applied to summarize the contribution of controlling factors to the deadwood mass loss. Here, microclimate (air temperature and relative humidity), soil conditions (soil pH, total soil nitrogen, and soil carbon-to-nitrogen ratio), and decomposer invertebrate activity (invertebrate exclusion effect) were set to affect deadwood mass loss rate. It was also assumed that microclimate influenced soil conditions and decomposer invertebrate activity. The fitness of the structural equation models was confirmed based on a chi-squared test (*P* > 0.05) and comparative fit index (> 0.90). Z-transformation was used for normalization of all variables. The structural equation models were conducted with the Lavaan package in R v.4.0.3 software (R Core Team, 2020).

Analysis of covariance and linear regression tests were implemented to show the univariate relationship between deadwood mass loss and the major controlling factors for each tree species (n = 9 for each tree species; *P* < 0.05). This analysis especially focused on the factors that had the highest path coefficient for either pine or oak deadwood mass loss in the structural equation model. The analysis of covariance and linear regression were conducted with the proc glimmix procedure in SAS v.9.4 software (SAS Institute Inc., USA).
